# Exposure to Extremely Low-Frequency Electromagnetic Fields Modulates Na^+^ Currents in Rat Cerebellar Granule Cells through Increase of AA/PGE_2_ and EP Receptor-Mediated cAMP/PKA Pathway

**DOI:** 10.1371/journal.pone.0054376

**Published:** 2013-01-22

**Authors:** Yan-Lin He, Dong-Dong Liu, Yan-Jia Fang, Xiao-Qin Zhan, Jin-Jing Yao, Yan-Ai Mei

**Affiliations:** Institutes of Brain Science, School of Life Sciences and State Key Laboratory of Medical Neurobiology, Fudan University, Shanghai, China; University of Sydney, Australia

## Abstract

Although the modulation of Ca^2+^ channel activity by extremely low-frequency electromagnetic fields (ELF-EMF) has been studied previously, few reports have addressed the effects of such fields on the activity of voltage-activated Na^+^ channels (Na_v_). Here, we investigated the effects of ELF-EMF on Na_v_ activity in rat cerebellar granule cells (GCs). Our results reveal that exposing cerebellar GCs to ELF-EMF for 10–60 min significantly increased Na_v_ currents (*I*_Na_) by 30–125% in a time- and intensity-dependent manner. The Na_v_ channel steady-state activation curve, but not the steady-state inactivation curve, was significantly shifted (by 5.2 mV) towards hyperpolarization by ELF-EMF stimulation. This phenomenon is similar to the effect of intracellular application of arachidonic acid (AA) and prostaglandin E_2_ (PGE_2_) on *I*_Na_ in cerebellar GCs. Increases in intracellular AA, PGE_2_ and phosphorylated PKA levels in cerebellar GCs were observed following ELF-EMF exposure. Western blottings indicated that the Na_V_ 1.2 protein on the cerebellar GCs membrane was increased, the total expression levels of Na_V_ 1.2 protein were not affected after exposure to ELF-EMF. Cyclooxygenase inhibitors and PGE_2_ receptor (EP) antagonists were able to eliminate this ELF-EMF-induced increase in phosphorylated PKA and *I*_Na_. In addition, ELF-EMF exposure significantly enhanced the activity of PLA_2_ in cerebellar GCs but did not affect COX-1 or COX-2 activity. Together, these data demonstrate for the first time that neuronal *I*_Na_ is significantly increased by ELF-EMF exposure *via* a cPLA2 AA PGE_2_ EP receptors PKA signaling pathway.

## Introduction

A number of studies have noted that exposure to extremely low-frequency electromagnetic fields (ELF-EMF) alter animal behaviors and modulate biological effects, including changes in gene expression, regulation of cell survival and promotion of cell differentiation [1]–[3]. In addition, exposure to ELF-EMF induces changes in cerebral blood flow in old Alzheimer’s mice. Enzyme activity in the cytosol or at the membrane and subsequent alternations in intracellular signaling are found in lymphoma B cells and Chinese hamster lung (CHL) cells upon exposure to ELF-EMF [4]–[6]. ELF-EMF can also modify the biophysical properties of cell membranes, such as changes in the membrane permeability of carbonic anhydrase [7], stimulation of the activity of Ca^2+^-activated potassium channels via increases in Ca^2+^ concentration [3], [8] and increased the expression level of Ca^2+^ channel protein [9]. However, very few studies have investigated the effects of EMF on sodium channels, in particular, the voltage-gated sodium (Na_v_) channels which are highly expressed in neurons.

Voltage-gated sodium channels (Na_V_) are one of the primary classes of ion channels responsible for driving neuronal excitability in both the central and peripheral nervous system. Na_V_ are clinically important because they play an important role in the generation of neuronal activity and alterations in Na_V_ are key factors in a number of pathologies [10]. Recent studies have revealed that Na_V_ channels participate in the rising phase of the neuronal action potential and contribute to many cellular functions including apoptosis, motility and secretory membrane activity [11]–[13]. Moreover, the EMF exposure was recently reported to modulate neuronal excitation and neurogenesis, which may be related to Na_V_ channel activity [8], [14], [15]. Thus, a thorough investigation of the influence of ELF-EMF on Na_V_ channels and the corresponding mechanism of action could help to uncover the effects of ELF-EMF-induced biological effects on brain physiology, pathogenesis and neural development.

Cerebellar granule cells (GCs) occupy a key position in the cerebellar–cortical circuitry by forming the input layer of the major cerebellar afferent system. Cerebellar GCs grown in primary culture express tetrodotoxin (TTX)-sensitive Na_V_ channels which are responsible for action potentials (APs) and for the code relay in the cerebellar circuitry [16], [17]. Cerebellar GCs are widely used as a model for neuronal cell development and apoptosis [18]–[20]. We have previously shown that the *I*_Na_ densities of cerebellar GCs are modulated by the lipid products ceramide and arachidonic acid (AA). Ceramide reduces the *I*_Na_ of cerebellar GCs by increasing calcium release through the ryanodine-sensitive Ca^2+^ receptor [21] while elevation in intracellular AA levels increases the *I*_Na_ of cerebellar GCs through the PGE_2_-mediated activation of the cAMP/PKA pathway [22]. The present study was conducted to determine whether exposure to ELF-EMF influences the Na^+^ channels of cerebellar GCs and, if so, whether this effect is mediated by changes in ceramide and/or arachidonic acid. The data presented in this report demonstrate that the activity of neuronal Na^+^ channels is significantly increased by ELF-EMF stimulation. Notably, the effect of ELF-EMF is mediated by an increase in cPLA_2_ activity and subsequent changes in intracellular concentration of arachidonic acid (AA) and EP receptor-mediated activation of the cAMP/PKA signaling pathway are involved.

## Results

**Figure 1:**
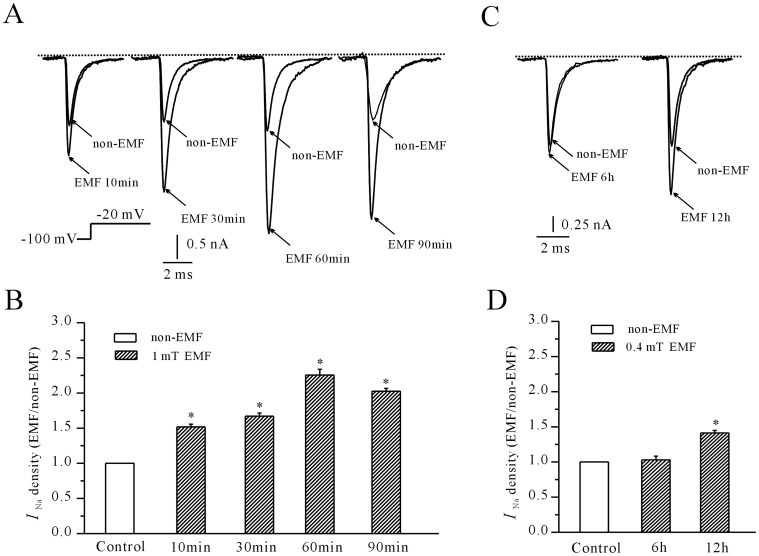
Time-dependent increase in *I*_Na_ in cerebellar GCs following exposure to ELF-EMF (1 mT or 0.4 mT). Superimposed *I*_Na_ evoked by a 20 ms depolarizing pulse from a holding potential from −100 to −20 mV. Current traces were obtained from cerebellar GCs exposed to ELF-EMF (1 mT) for lengths of time ranging from 10 min to 90 min. (B) Statistical analysis of the activating effects of ELF-EMF (1 mT) exposure at various times on the density of *I*_Na_. The data are reported as the mean ± S.E.M. from 8–16 cells. *, *P*<0.05 compared to control using a one-way ANOVA test. (C) *I*_Na_ traces obtained from cerebellar cells exposed to ELF-EMF (0.4 mT) for 6 h and 12 h. (D) Statistical analysis of the activating effects of ELF-EMF (0.4 mT) exposure for 6 h and 12 h on the density of *I*_Na_. The data are reported as the mean ± S.E.M. from 8–10 cells. *, *P*<0.05 compared to control using Student’s *t*-test.

First, we investigated the actions of extremely low frequency electromagnetic fields (ELF-EMF) on the *I*_Na_ of cerebellar GCs. An *I*_Na_ was elicited by a depolarization step to −20 mV from the holding potential of −100 mV. When cerebellar GCs were exposed to ELF-EMF (1 mT) for 10 min, the amplitude of the *I*_Na_ was increased by approximately 51.8% ±3.8% (*n* = 12, *P*<0.05) compared to cells that were not exposed to ELF-EMF (*n* = 8, Fig. 1A). However, the mean capacitance of recorded cells for the control group (13.05±0.40 pF, *n* = 7) and for the ELF-EMF treatment group (12.45±0.77pF, *n* = 6) showed no significant difference (*P* = 0.48). The similar in capacitance indicated that the increased current density upon ELF-EMF exposure was not due to different in abnormal cell morphology. In addition, the increase in *I*_Na_ density induced by ELF-EMF exposure was time dependent. When cerebellar GCs were exposed to ELF-EMF for 30 min, 60 min or 90 min, the density of the *I*_Na_ increased by 67.1% ±4.38% (*n* = 8, *P*<0.05), 125.6% ±8.26% (*n* = 16, *P*<0.05), 102.4% ±4.1% (*n* = 9, *P*<0.05), respectively, compared to controls (Fig. 1B). We also tested the effects of low-density ELF-EMF (0.4 mT) on *I*_Na_. The results shown in Fig. 1C indicated that, when cerebellar GCs were exposed to 0.4 mT ELF-EMF, a longer exposure time was needed to induce an increase in *I*_Na_ density. The density of *I*_Na_ was only increased by 3.62% ±2.38% (*n* = 6) when cerebellar GCs were exposed to ELF-EMF (0.4 mT) for 6 h. Upon an increase in the length of cell exposure to ELF-EMF (0.4 mT) to 12 h, the *I*_Na_ density increased by 31.6% ±3.38% (*n* = 8, *P*<0.05, Fig. 1D). Because a previous study indicated that when cells are exposed to ELF-EMF over a long term, their levels of protein expression may be affected [5], which will be difficult to further identify the primary factor involved in the ELF-EMF-induced *I*_Na_ increase. Thus, we chose to focus on the mechanism by which a relatively short-term exposure to a 1 mT ELF-EMF induces an *I*_Na_ increase.

**Figure 2:**
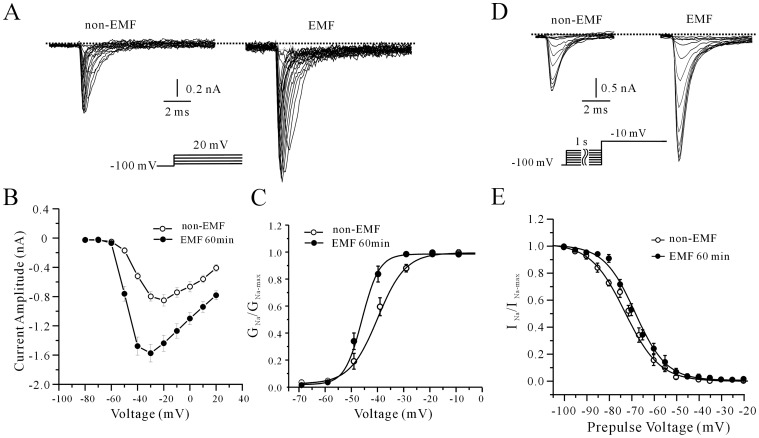
The effects of 1 mT ELF-EMF exposure on the steady-state activation and inactivation of *I*_Na._ (A) The effects of 60 minutes of ELF-EMF exposure on the steady-state activation of *I*_Na_. The cells were held at −100 mV and depolarized in 5 mV steps from −70 to 20 mV with intervals of 5 s. (B) The voltage-dependent activation curve of *I*_Na_ in control cells and cells exposed to ELF-EMF. The data are from 13 (control) or 12 (ELF-EMF-exposed) cells and are expressed as means ± SEM. (C) Comparison of the plot of the normalized conductance of *I*_Na_ as a function of the command potential in control and ELF-EMF-exposed cells. (D) The effects of 60 minutes of ELF-EMF exposure on the steady-state inactivation of *I*_Na_. The voltage protocol is shown below the current record. (E) Steady-state inactivation curves of *I*_Na_ for control and ELF-EMF-exposed cells. The data are from 6 (control) and 5 cells (ELF-EMF-exposed) and are expressed as means ± S.E.M.

Next, we investigated the effects of ELF-EMF on the voltage-gating properties of *I*_Na_ channels. The activation properties of *I*_Na_ in cerebellar GCs following exposure to ELF-EMF were studied using the appropriate voltage protocols. *I*_Na_ were evoked by a 20 ms depolarizing pulse from a holding potential of −100 mV to potentials between −70 and 20 mV, with 5 mV steps 5 s intervals (Fig. 2A). Fig. 2B illustrated a voltage-current curve in which the maximum activation potential was changed from −27.3±0.9 mV to −32.5±2.5 mV when cerebellar GCs were exposed to ELF-EMF for 60 min (*n* = 13 for control and *n* = 12 for ELF-EMF exposure, *P*<0.05). A value for steady-state activation of *I*_Na_ was then obtained by normalizing the conductance as a function of the command potential; conductance was calculated as *G*_Na_ = *I*_Na_/(Vm1/2–Vrev). The data points were fitted to the Boltzmann function *G*_Na_/*G*_Na_-max = 1/{1+exp [(Vm1/2–Vm)/k]}. As shown in Fig. 2C, after cerebellar GCs were exposed to ELF-EMF for 60 min, the half-activation potentials changed from −40.93±0.67 mV to −46.87±1.3 5 mV (*n* = 10, *P*<0.05 ), with slope factors of 3.7±0.1 mV and 4.6±1.0 mV (*n* = 10). These data indicate that ELF-EMF exposure significantly shifts the voltage dependence of the steady-state activation of *I*_Na_ of cerebellar GCs.

Next, we studied the effects of ELF-EMF exposure on the voltage dependence of the steady-state inactivation of *I*_Na_ channels. *I*_Na_ were elicited using 1-s conditioning pre-pulses ranging from −100 to −40 mV in steps of 5 mV prior to a −10 mV test pulse (Fig. 2D). The steady-state inactivation curve was then fitted using the Boltzmann equation *I*_Na/_ *I*_Namax_ = 1/{1+exp [(*V*_m_–*V*_m1/2_)/*k*]} +*A*. In the 11 cells studied, the *V*_m 1/2_ values were −72.7±0.74 mV (*n* = 6) and −68.2±0.75 mV (*n* = 5) for cells with or without ELF-EMF exposure, respectively (Fig. 2E). These data indicate that the steady-state inactivation curve of *I*_Na_ in cerebellar GCs did not significantly shift upon exposure to ELF-EMF.

**Figure 3:**
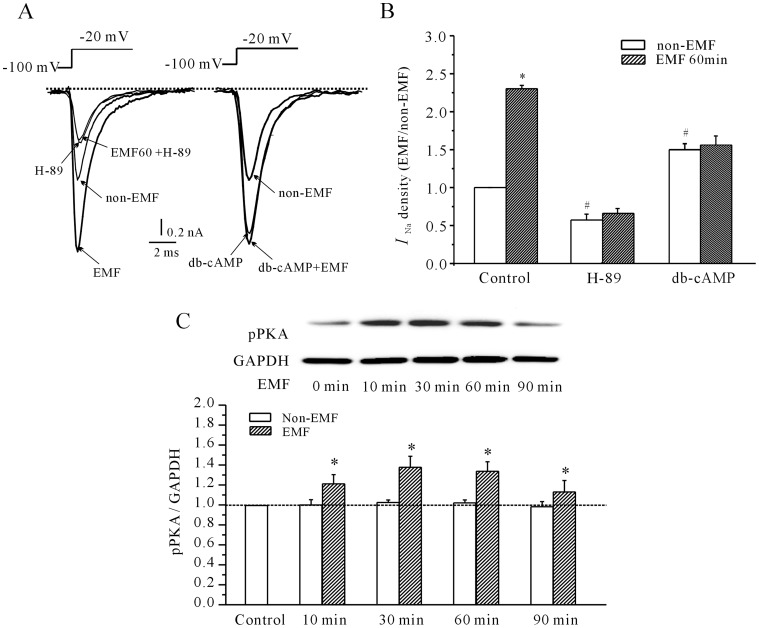
Involvement of the PKA pathway in the increase in *I*_Na_ induced by ELF-EMF exposure. (A) Current traces show the inhibitory effects of the selective PKA antagonist H-89 and db-cAMP on the increase in *I*_Na_ induced by a 60-minute exposure to a 1 mT ELF-EMF. (B) Statistical analysis of the inhibitory effects of H-89 and db-cAMP on the increase in *I*_Na_ density induced by a 60-minute exposure to a 1 mT ELF-EMF. The data are reported as the mean ± S.E.M. from 10–12 cells. *, *P*<0.05 compared to control (non-ELF-EMF group) using a Student’s *t*-test. #, *P*<0.05 compared to the corresponding control (without H-89 and db-cAMP) non-ELF-EMF group using a Student’s *t*-test. (C) Western blot analysis of the effects of ELF-EMF exposure on cellular pPKA levels. Upper panels show representative samples; the statistical analysis is shown in the lower panels; *, *P* < 0.05 compared to the corresponding control using Student’s *t*-test.

Our previous study showed that elevation in arachidonic acid (AA) concentration increased *I*_Na_ through prostaglandin E_2_ (PGE_2_)-mediated activation of the cAMP/PKA pathway [22]. In the present study, the effect of ELF-EMF exposure on *I*_Na_ and the *I*_Na_ steady-state activation curve was similar to the effect of AA on *I*_Na_. We therefore tested whether cAMP/PKA activation was involved in the increase in *I*_Na_ in cerebellar GCs subjected to ELF-EMF exposure. The results in Fig. 3A showed that H-89, a selective PKA antagonist, significantly attenuated the increase in *I*_Na_ elicited by ELF-EMF exposure. Incubating cerebellar GCs with 10 µM H-89 for 60 minutes decreased the evoked *I*_Na_ by 42.7% ±4.0% (*n* = 9, *P*<0.05). There was, however, only 12.4% ±6.4% (n = 14) in *I*_Na_ after exposure to ELF-EMF in the presence of H89. Hence, H89 suppressed the induction of the *I*_Na_ activity after ELF-EMF exposure. Similarly, administration of 10 µM dibutyryl cAMP (db-cAMP, a embrane-permeable cAMP analog) produced a significant increase of *I*_Na_ by 47.2±6.6% (*n* = 7, *P*<0.05). Notably, exposure to ELF-EMF did not further potentiate the effect of db-cAMP on *I_Na_* (54.8±8.9%, *n* = 20). Hence, these data support our conclusion that cAMP/PKA pathway is involved in the effect of ELF-EMFs on *I*_Na_.

In addition, we determined the intracellular levels of phosphorylated PKA (pPKA) by immunoblot assays and found significant increase in phospho-PKA after ELF-EMF exposure. These data indicate that when the cells were exposed to ELF-EMF, the intracellular levels of pPKA were increased by 38.6% ±3.76% (*n* = 4, *P*<0.05), 42.4% ±4.32% (*n* = 4, *P*<0.05),30.4% ±1.5% (*n* = 4, *P*<0.05) and 17.8±2.14% (*n* = 4, *P*<0.05), respectively (Fig. 3C). These data suggest that PKA activation is associated with the ELF-EMF-induced increase in *I*_Na_ in cerebellar GCs.

**Figure 4:**
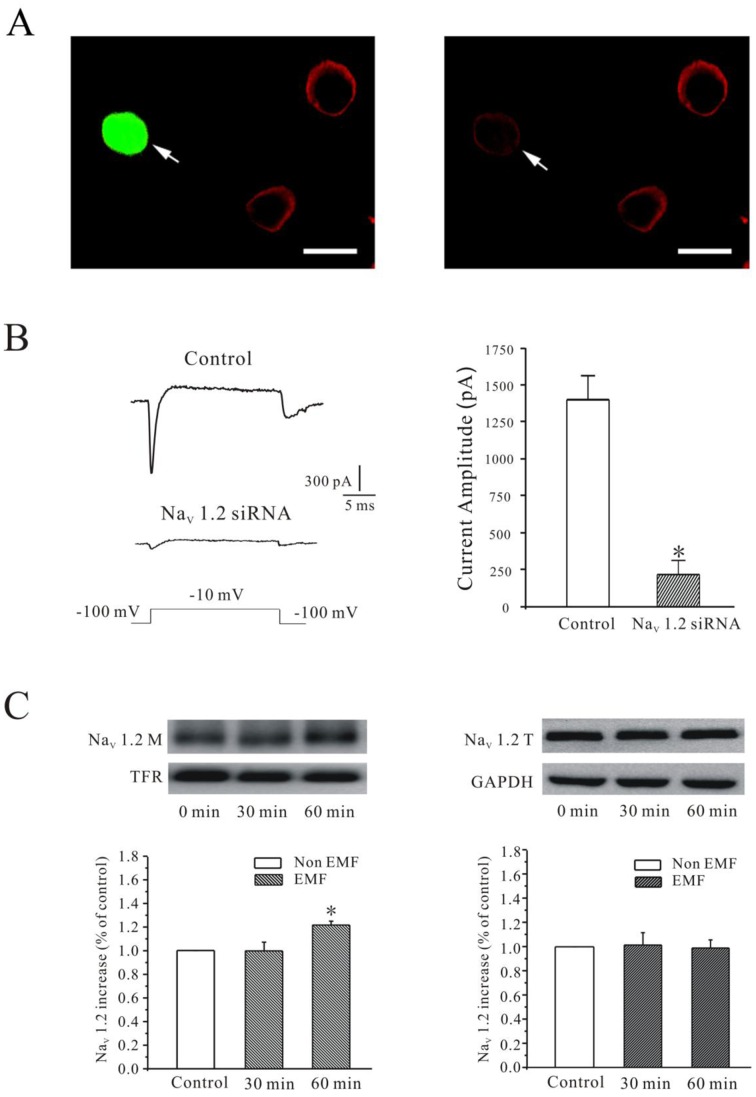
ELF-EMF exposure increased the Na_V_1.2 α-subunit on the membrane in cerebellar GCs. (A) Cells expressing Na_V_ 1.2 were labeled with Na_V_1.2 specific antibody (red one) and transfected GC cells showing strong eGFP expression (left, see arrowhead). Cells transfected with siRNA vectors (same cell in the right, see arrowhead) showed dramatic reduction in Na_V_ 1.2 expression. Scale bar was 10 µM. (B) The current recordings in a control cell and a post-transfected cell of Na_V_ 1.2 siRNA plus eGFP. Current evoked by a 20 ms depolarizing pulse from a holding potential of −100 to −20 mV. (C) Na_V_ 1.2 protein on membrane surface (Na_V_ 1.2 M) was detected with the biotinylation assay after 30 or 60-minute exposure to a 1 mT ELF-EMF. Upper panels show representative samples, TFR (transferrin) was used as the loading control; the statistical analysis is shown in the lower panels. *, *P*< 0.05 compared to the corresponding control using Student’s *t*-test. (D) Western blot analysis of the total level of Na_V_ 1.2 expression (Na_V_ 1.2 T) after 30 or 60-minute exposure to a 1 mT ELF-EMF.

Previous study indicated that Na_V_ activities can be modulated by the cAMP/PKA pathways upon phosphorylation of the α-subunits of Na_V_ channels [23]. Besides increases in the modification of steady-state activation propriety, exposure to ELF-EMF might also elicit insertion of additional Na_V_ channels into the membrane, which might then further account for the ELF-EMF-induced up-regulation of *I*_Na_. To investigate whether ELF-EMF induced insertion of new Nav channels on the membrane, we first ascertained that Na_V_ 1.2 α-subunits plays a significant role in Na_V_ channel of cerebellar GCs as reported previously [17]. As shown in Fig. 4A, the expression of Na_V_ 1.2 was significantly reduced by siRNA. Similarly, the current amplitude of *I*_Na_ was also significantly reduced. The current amplitude of *I*_Na_ recorded from the control group was −1400.32±162.15 pA (*n = *7), and it was reduced to −207.89±95.55 pA (*n* = 5, *P*<0.05) after knocking down the Na_V_ 1.2 gene (Fig. 4B).

Next, we determined the levels of Na_V_ 1.2 protein on membrane surface using biotinylation assay. The data obtained from four independent experiments showed that the Na_V_ 1.2 protein on the cerebellar GCs membrane (Na_V_ 1.2 M) was increased by 21.8% ±3.3% after exposed to ELF-EMF for 60 min (Fig. 4C). The total expression levels of Na_V_ 1.2 protein (Na_V_ 1.2 T), however, was not affected after exposure to ELF-EMF (Fig. 4D). These data indicate that exposure to ELF-EMF activates PKA, which then modulates *I_Na_*, in part, by insertion of new Na_V_ channels into the membrane.

**Figure 5:**
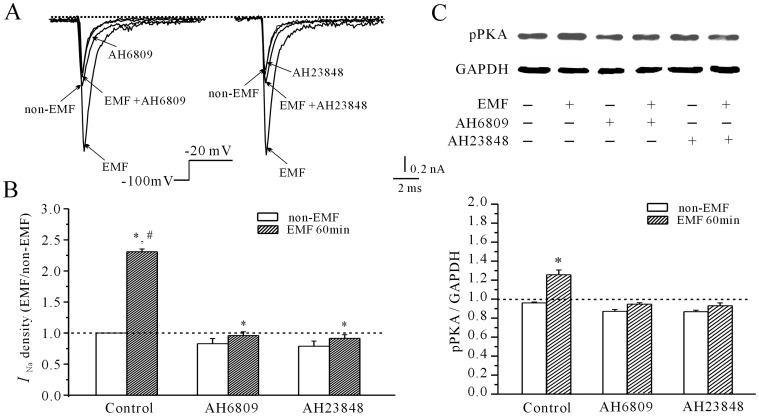
Effects of EP receptor antagonists on the increase in *I*_Na_ density induced by ELF-EMF exposure. (A) Current traces show the blocking effect of the EP2 and EP4 receptor antagonists AH6809 and AH23848 on the increase in *I*_Na_ induced by a 60-minute exposure to a 1 mT ELF-EMF. (B) Statistical analysis of the inhibitory effect of AH6809 and AH23848 on the *I*_Na_ density increase induced by a 60-minute exposure to a 1 mT ELF-EMF. #, *P*<0.05 compared to the corresponding control (non-ELF-EMF) using Student’s *t*-test. *, *P*<0.05 compared to ELF-EMF exposure alone using a Student’s *t*-test. (C) Western blot analysis of the inhibitory effect of AH6809 and AH23848 on intracellular PKA phosphorylation induced by a 60-minute exposure to a 1 mT ELF-EMF. Upper panels show representative samples; the statistical analysis is shown in the lower panels; *, *P*< 0.05 compared to the corresponding control using Student’s *t*-test.

It is well known that the cellular effects of PGE_2_ are mediated through a family of G-protein-coupled receptors designated EP1, EP2, EP3 and EP4 [24], [25]. Of these, EP2 and EP4 are involved in activation of the cAMP/PKA pathway [26]. Our previous study demonstrated that all four types of EP receptors mRNA are found in cerebellar GCs [22]. Because EP2 and EP4 can activate the PKA pathway, we used EP2 and EP4 receptor antagonists (AH6809 and AH23848, respectively) to investigate whether EP receptors played a role in the activation of PKA after exposure to ELF-EMF. As shown in Fig. 5A and 5B, in the presence of 20 µM AH6809 or 20 µM AH23848, 60 minutes of ELF-EMF exposure only increased *I*_Na_ by 9.54% ±4.7% (*n* = 6) and 5.52±4.71% (*n* = 5), respectively. In the absence of the EP receptor antagonists, exposure to ELF-EMF increased *I*_Na_ to 125.6% ±8.26% (*n* = 16, *P*<0.05). Similarly, blocking EP2 and EP4 receptors using AH6809 or AH23848 significantly reduced the ELF-EMF exposure-induced increase in pPKA levels. The results in Fig. 5C showed that in the presence of 20 µM AH6809 or 20 µM AH23848, 60 min of ELF-EMF exposure only increased intracellular levels of pPKA by 7.25% ±1.67% or 6.5% ±2.8% (*n* = 4), respectively.

**Figure 6:**
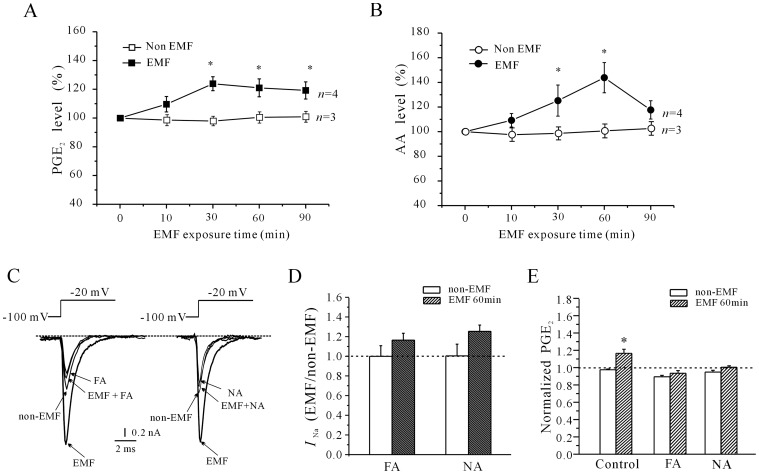
Effects of the AA/PGE_2_ pathway on the ELF-EMF exposure-induced increase in *I*_Na_ in cerebellar GCs. (A) PGE_2_ release from cerebellar GCs exposed to 1 mT ELF-EMF for various lengths of time. The data are from three and four independent experiments, respectively. *, *P*< 0.05 compared to the corresponding control using Student’s *t*-test. (B) Intracellular AA levels in cerebellar GCs exposed to 1 mT ELF-EMF for various lengths of time. The data are from three and four independent experiments, respectively. *, *P*< 0.05 compared to the corresponding control using Student’s *t*-test. (C) Current traces show the inhibitory effects of the COX-2 inhibitors flufenamaic acid (FA) and niflumic acid (NA) on the increase in *I*_Na_ induced by 60 minutes of exposure to a 1 mT ELF-EMF. (D) Statistical analysis of the effects of FA and NA on the increase in *I*_Na_ induced by a 60-minute exposure to a 1 mT ELF-EMF. (E) Statistical analysis of the effects of FA and NA on PGE_2_ release induced by a 60-minute exposure to a 1 mT ELF-EMF.

Because EP receptors, which stimulate the cAMP/PKA signaling pathway, are activated by PGE_2_, we next studied the release of PGE_2_ from cerebellar GCs following exposure to ELF-EMF using a direct rat PGE_2_ ELISA kit. As shown in Fig. 6A, the amount of PGE_2_ released from control cerebellar GCs was 64.36±1.88 pmol. Following exposure to ELF-EMF for 10 min, 30 min, 60 min or 90 min, the PGE_2_ content obtained from three independent experiments was significantly increased by 9.6% ±5.3%, 23.9% ±4.8%, 20.9% ±6.2% and 19.2% ±5.9%, respectively (*n* = 3, *P*<0.05). We also assessed the levels of intracellular AA, which can be metabolically converted to PGE_2_, using a direct rat AA ELISA kit to address whether the increase in PGE_2_ after ELF-EMF exposure was the result of an increase in intracellular AA. The results in Fig. 6B showed that the intracellular AA levels were significantly increased following exposure to ELF-EMF. When cerebellar GCs were exposed to ELF-EMF for 10 min, 30 min, 60 min or 90 min, the intracellular AA levels measured in three independent experiments were significantly increased by 9.25±5.45%, 25.21±1.26%, 43.8±1.23% and 17.64±7.4%, respectively (*n* = 4, *P*<0.05).

AA is converted to prostaglandins by either the cyclooxygenase, the lipoxygenase or the monooxygenase pathways [27]. In particular, cyclooxygenase-2 (COX-2) is reported to play a key role in the release of PGE_2_ [28]. To test that the increase in *I*_Na_ after ELF-EMF exposure was due to an increase in intracellular AA, we investigated the effect of the selective COX-2 inhibitors flufenamaic acid (FA) and niflumic acid (NA). Administration of 10 µM FA or 20 µM NA alone reduced the *I*_Na_ amplitude of cerebellar GCs by 23.6% ±1.2% (*n* = 4) and 14.7% ±2.3% (*n* = 8), respectively. In the presence of 10 µM FA or 20 µM NA, the increase in *I*_Na_ induced by ELF-EMF exposure was abolished (Fig. 6C and 6D). The *I*_Na_ density in cerebellar GCs exposed to ELF-EMF for 60 min was increased by only 14.3±3.4% (*n* = 8) or 6.3±2.7% (*n* = 10), respectively, which were significantly different from those derived from cerebellar GCs exposed to ELF-EMF alone (125.6% ±8.26%, *n* = 16). Similarly, FA or NA treatment abolished the increase in PGE_2_ release induced by ELF-EMF exposure (Fig. 6E). Data obtained from three independent experiments showed that the release of PGE_2_ from cerebellar GCs exposed to ELF-EMF for 60 min was only increased by 3.3% ±3.0% and 2.6±1.5% in the presence of FA or NA, respectively.

**Figure 7:**
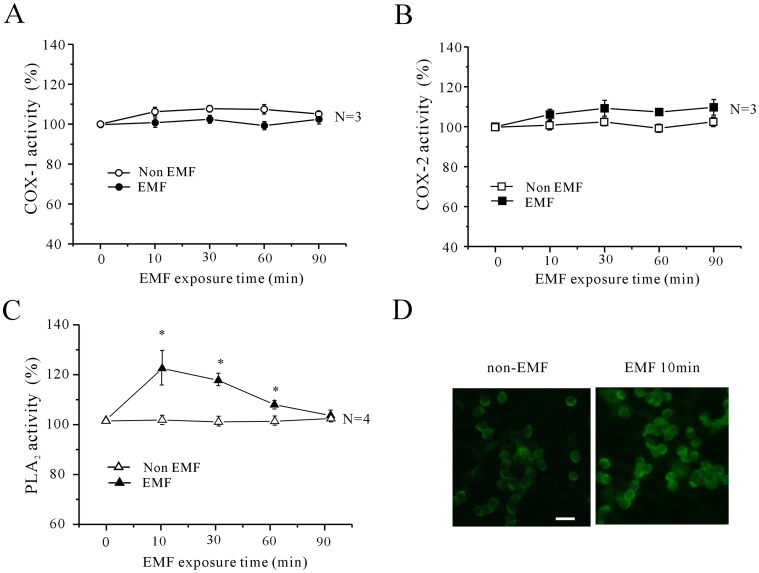
Effects of exposure to a 1 mT ELF-EMF on intracellular COX-1, COX-2 and cPLA_2_ activities in cerebellar GCs. (A and B) COX-1 and COX-2 activity were measured in cerebellar GCs exposed to a 1 mT ELF-EMF for various lengths of time. The data are from three and four independent experiments, respectively. (C) cPLA_2_ activity was measured in cerebellar GCs exposed to a 1 mT ELF-EMF for various lengths of time. *, *P<*0.05 compared to the corresponding control using a Student’s *t*-test. (D) Immunostaining showing the effects of ELF-EMF exposure on intracellular cPLA_2_ levels. The scale bar represents 20 µm.

To ascertain the role of COX in mediating the effects of ELF-EMF on cerebellar GCs, we determined the activities of both COX-1 and COX-2 using selective COX-1 and COX-2 ELISA kits. When cerebellar GCs were exposed to ELF-EMF for lengths of time ranging from 10 min to 90 min, the intracellular activity of COX-1 was increased from 5.0±1.2% to 6.3±2.2% (Fig. 7A) while the activity of COX-2 was increased from 6.1±2.6% to 9.7±3.9% (Fig. 7B). Hence, neither COX-1 nor COX-2 activity was significantly different than non-ELF-EMF-exposed cells. These data suggest that the increase in PGE_2_ release after exposure to ELF-EMF was due to an increase in intracellular AA rather than in COX enzyme activity.

**Figure 8:**
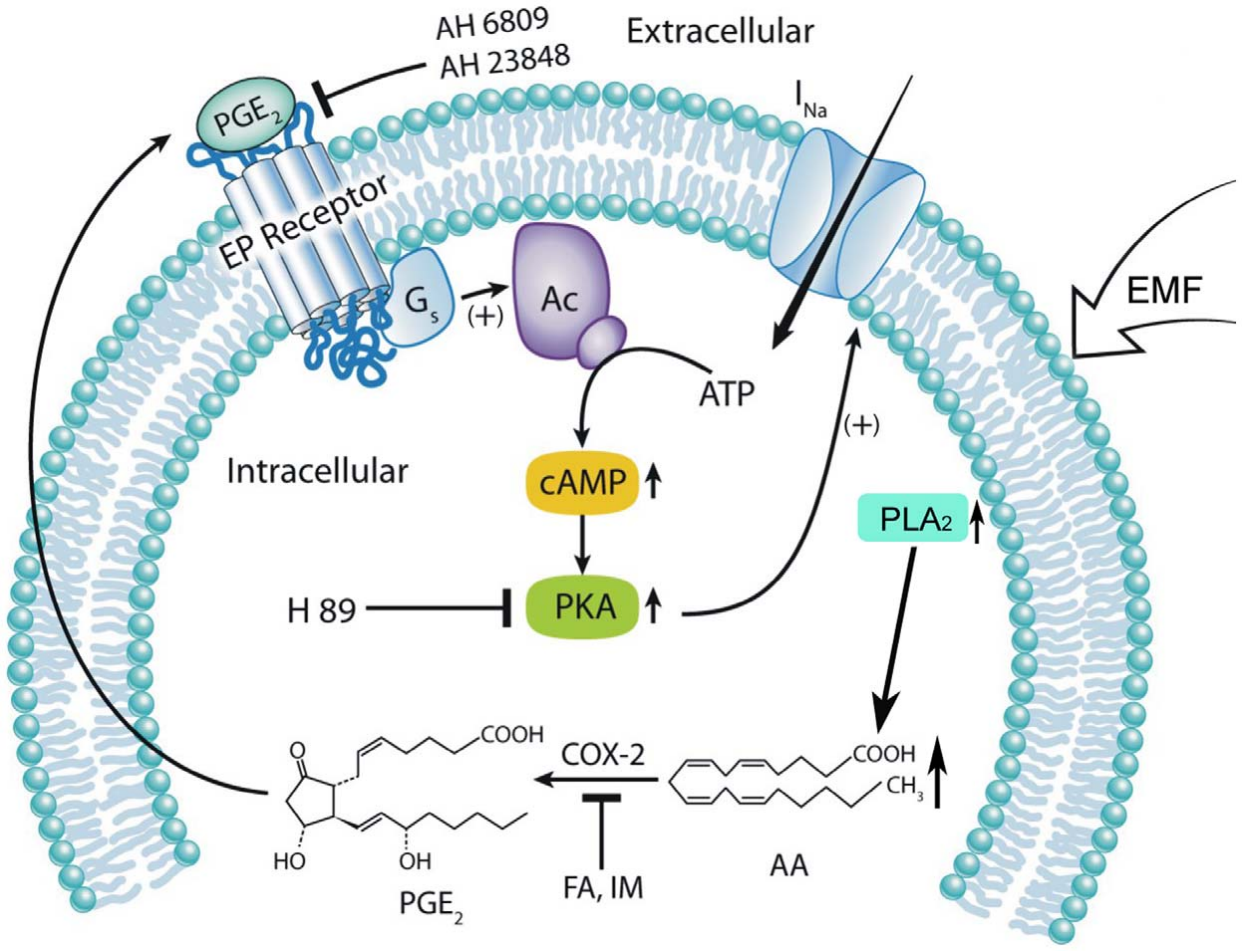
A proposed model depicting the mechanisms that are likely to be involved in the modulation of *I*_Na_ by ELF-EMF exposure in cerebellar GCs. ELF-EMF active cPLA_2_ and up-regulated AA and PGE2, which can act in an autocrine or paracrine manner to activate EP receptors. Ligand binding of EP2/4 is associated with PKA activation and consequently modulates *I*_Na_. (+), activation; (−), inhibition.

Given that cytosolic phospholipase A_2_ (cPLA_2_) also plays an important role in producing intracellular AA [29], we measured the activity of intracellular cPLA_2_ in cerebellar GCs before and after ELF-EMF exposure using an ELISA kit and immunocytochemistry. As shown in Fig. 7C, ELF-EMF exposure significantly enhanced cPLA_2_ activity, especially in the first 10 min to 30 min after exposure. Data obtained from four independent experiments indicated that exposing cerebellar GCs to ELF-EMF for 10 min, 30 min, 60 min or 90 min significantly increased cPLA_2_ activity by 19.78±6.93%, 15.25±2.7%, 5.92±1.61% and 1.76±2.0%, respectively (*n* = 4, *P*<0.05). The effects of ELF-EMF exposure on intracellular cPLA_2_ levels in cerebellar GCs were confirmed by immunostaining. As shown in Fig. 7D, a low level of cPLA_2_ labeling was detected in cerebellar GCs without exposure to ELF-EMF (Fig. 7D, left). After exposure to ELF-EMF, cerebellar GCs showed a significant increase in staining for cPLA2 (Fig. 7D, right), indicating that ELF-EMF exposure significantly increased intracellular cPLA_2_ levels. A model depicting the possible mechanisms involved in the modulation of *I*_Na_ by ELF-EMF exposure in cerebellar GCs is shown in Fig. 8.

## Discussion

Although ELF-EMF exposure has been previously reported to modulate the activity of ion channels, few studies to date have measured the effects of EMF exposure on neuronal *I*_Na_. Here, we report for the first time that ELF-EMF exposure enhances *I*_Na_ in cerebellar GCs. In particular, exposure of cerebellar GCs to ELF-EMF influences the activity of PLA_2_, thus stimulating the production of intracellular AA, which is then converted to PGE_2_. PGE_2_ then enters the extracellular space and binds to EP receptors, activating the cAMP/PKA pathway and accounts for the induction of *I*_Na_.

### ELF-EMF Increases I_Na_ in Rat Cerebellar GCs and Alters Na_V_ Channel Kinetics

Although several ion channels, such as Ca^2+^ and Ca^2+^-activated K^+^ channels [8], [9], are known to be modulated by EMF, the effects of EMF on Na_V_ channels are poorly understood. Arthur D. Rosen’s studies in GH3 cells indicated that there was a slight shift in the current-voltage relationship and a less than 5% reduction in peak current during 125 mT static magnetic field exposure [30].The authors speculated that a physical deformation in the liquid crystal membrane may be associated with these changes [30]. However, the changes observed in the steady-state activation characteristics of Na_V_ channel kinetics during exposure to ELF-EMF in our study are due to an AA metabolite-induced increase in *I*_Na_, a result that is similar to those obtained previously [22]. Interestingly, the effects of ELF-EMF on carbonic anhydrase entrapped in liposomes seem to exclude the role of protein molecules but in favor of a direct action on charged lipids of the membrane [7]. Moreover, in experiments using Ca^2+^-activated K^+^ channels in dorsal root ganglion-isolated neurons, Marchionni et al. did not observe modifications in single-channel behavior that suggested a direct action of EMF on membrane proteins [8], consistent with the hypothesis that ELF-EMF-stimulated enhancement of *I*_Na_ in rat cerebellar GCs is not related directly to physical changes within the membrane and that an AA metabolism-related pathway might be involved. The discrepancy of these two reports may be due to the use of different cell type and different form of EMF source.

The time course for the effects of ELF-EMF on the activity of Na_V_ in this study is noteworthy. In previous studies, the effects of exposure to EMF differed significantly based on whether the exposure occurred for minutes, hours or days. Here, we demonstrate that exposure to ELF-EMF induced similar effects on *I*_Na_ in rat cerebellar GCs regardless the condition is 1 mT stimulation for a short time or 0.4 mT stimulation for a longer time. Notably, it is generally believed that short-term changes induced by EMF are mediated by modifications in enzyme activity in the cytosol or the membrane [4], [31], [32] while the long-term exposure to EMF may induce changes in nuclear functions such as gene transcription and cell cycle regulation [33], [34]. Moreover, a previous study in cerebellar GCs indicated that a five-day EMF exposure contributes to premature expression of GluRs, and leads to more rapid cellular maturation [35]. To avoid the influence of multiple factors due to long-term EMF exposure, we performed all our experiments at 1 mT EMF exposure for a short time, which we believe it is well-suited to access the effect of ELF-EMF on intracellular signaling pathways.

### ELF-EMF Exposure Enhances I_Na_ via PGE_2_-mediated Activation of the cAMP/PKA Pathway

It has been reported that the cAMP/PKA pathway can modulate Na_V_ activity. In particular, the α-subunits of Na_V_ channels are preferred substrates for phosphorylation by cAMP/PKA [36]. Either activation or inhibition of Na_V_ channels has been reported, which is probably due to the difference in cell model used [37], [38]. Our previous study demonstrated that intracellular application of AA increases the activity of Na_V_ via the activation of the cAMP/PKA pathway in cerebellar GCs [22]. Consistent with the previous findings, we observed that the PKA signaling plays an important role in the increase in *I*_Na_ after exposure to ELF-EMF. In light of our findings that ELF-EMF activates PKA signaling (Fig. 3), modifies steady state properties of the Na_V_ (Fig. 2) and increases insertion of Na_V_ into the membrane (Fig. 4), we speculate that the effect of ELF-EMFs on *I*_Na_ is likely exerted at multiple levels. In addition to the changes in voltage-gating property and insertion of additional Na_V_ channels into the membrane, exposure to ELF-EMFs may also modulate the single channel properties by phosphorylation of channel protein. Recording of single-channel activity, however, is of technically challenging and requires specialized set up for such precise measurement. Nonetheless, the possible regulation of Na_V_ channels upon PKA phosphorylation warrants future investigations.

Previous studies by Sibylle Thumm et al. reported that ELF-EMF (20 Hz, 7–8 mT) exposure for 60 min resulted in an increase in PKA activity in human skin fibroblasts and rat embryonic osteoblasts [39]. The underlying mechanism by which PKA was activated by ELF-EMF exposure, however, was not known. In synovial fibroblasts of an osteoarthritis patient, ELF-EMF exposure-induced changes in cAMP levels were associated with a selective increase in adenosine receptor expression [33]. Similarly, our results indicate that exposure to ELF-EMF induces an increase in PKA activity and that treatment with EP2 and EP4 antagonists abolished this effect. Moreover, we observed that, along with EP receptor activation, PGE_2_ levels were also increased following ELF-EMF exposure. Our data indicate that the effects of ELF-EMF exposure on PKA activity might not reflect a direct effect on this enzyme. Rather, PKA activation is mediated by elevations of PGE_2_, which then binds to the EP receptors to modulate PKA activity.

### Increases in Intracellular PLA_2_ Activity and AA Levels are Critical Steps in the ELF-EMF-mediated Enhancement of I_Na_

It is well known that intracellular PGE_2_ is derived from AA via the cyclooxygenase (COX) pathway, which includes COX-1 and COX-2 [28], and COX-2 has been reported to be the most abundant COX isozyme in neurons [40]. In our study, intracellular PGE_2_ and AA levels were increased in cerebellar GCs following ELF-EMF exposure. Neither COX-1 nor COX-2 activity was significantly enhanced by ELF-EMF exposure. Inhibiting COX activity with specific inhibitors, however, eliminated the ELF-EMF exposure-induced increase in *I*_Na_. Hence, an alternative pathway is likely involved for the increase in intracellular PGE_2_ levels after ELF-EMF exposure.

In brain tissue, AA is mainly released downstream of cPLA_2_. In this study, our results showed that intracellular AA levels in cerebellar GCs were significantly increased after EMF exposure. cPLA_2_ activity, which is important for rapid AA release in neurons, was also significantly enhanced by exposure to ELF-EMF, suggesting that the increases in cPLA2 activity and AA levels are critical steps in the ELF-EMF-induced enhancement of *I*_Na_. Using a cell-free PLA_2_ assay, Song et al. failed to observe changes in PLA_2_, including cPLA_2_ and sPLA_2_, following exposure to ELF-EMF (60 Hz, 0.1 or 1 mT) for 4 or 6 h [41]. The difference in these findings compared to our results in cerebellar GCs could be due to the use of a cell-free PLA_2_ assay as cell membranes play an important role in mediating the effects of EMF on enzymatic activity [31]. Despite there is evidence for the involvement of cPLA2 activity in the responses to a wide variety of stimuli such as oxidative stress and inflammatory factors [42], [43], the relevant mechanism of ELF-EMF exposure-induced cPLA_2_ activation remains unclear.

### Physiological Implications

The effects of ELF-EMF on nerve cells have been extensively studied in various organisms [8], [35]. Although the reported results are variable or contradictory due to differences in the experimental conditions and in the density and/or duration of EMF exposure, EMF has recently been reported to modulate of neuronal excitatory functions and neurogenesis [8], [14], [15]. In addition, recent studies have revealed an important role for cPLA2 in the modulation of neuronal excitatory functions [29]. The findings from our molecular-level analysis of the macroscopic effects on *I*_Na_ produced by exposure to ELF-EMF provide evidence for an important effect of EMF on neuronal excitation in the CNS. We also observed that the effects of ELF-EMF exposure on cerebellar GCs peaked and then declined with extended exposure time. Notably, this phenomenon was observed for *I*_Na_ and also for enzyme activities. The observed effects is likely due to the cells reach equilibrium through self-regulation after the initial ELF-EMF exposure and its effect on cPLA_2_ and *I*_Na_. Nonetheless, the modulatory effects on neuronal excitatory caused by exposure to ELF-EMF are complicated and varied. Therefore, further exploration is required to comprehensively analyze the physiological and/or pathological effects of ELF-EMF exposure on *I*_Na_, along with the resulting neuronal consequences.

## Materials and Methods

### Ethics Statement

This study was carried out in strict accordance with the recommendations in the Guide for the Care and Use of Laboratory Animals of the National Institutes of Health. The protocol was approved by the Committee on the Ethics of Animal Experiments of the Fudan University (Permit Number: 20090614-001). All surgery was performed under sodium pentobarbital anesthesia, and all efforts were made to minimize suffering.

### Primary Cell Culture

Cells were derived from cerebellum of 7-day-old Sprague–Dawley rat pups as described previously [44]. Isolated cells were then plated onto 35-mm-diameter Petri dishes coated with poly-L-lysine (1 µg/ml) at a density of 2.5×10^5^/cm^2^. Cultured cells were incubated at 37°C with 5% CO_2_ in Dulbecco’s Modified Eagle’s Medium (DMEM) supplemented with 10% fetal calf serum, glutamine (5 mM), insulin (5 µg/ml), KCl (25 mM), and 1% antibiotic–antimycotic solution. All experiments were carried out with cerebellar GCs during 6–8 days in culture (DIC).

### Electromagnetic Field Production

The system used to expose cerebellar GCs cells to electromagnetic fields was the same used in previous studies, with some revisions (I-ONE, Shanghai, China) [33], [45], [46]. Briefly, a 50 Hz magnetic field was generated by a pair of Helmholtz coils placed in opposition to each other. The coils were powered by a generator system that produced the input voltage of the pulse, and the magnetic flux densities could be regulated within the range of 0 to 1.0 mT. The device was powered by an AC power generator, and the EMF frequency and density were monitored by an EMF sensor that was connected to a digital multimeter. The geometry of the system assured a uniform field for the exposed cultured cells. The surfaces of the culture plates were parallel to the force lines of the alternating magnetic field in the solenoid. Air and culture medium temperatures were continuously monitored for the duration of experiments. The maximum temperature increase recorded in the cultures that were exposed to ELF-EMF (compared to non-exposed cultures) was 0.4±0.1°C. To identify any possible influence of this increase on our results, we compared data obtained from cerebellar GCs cultured in two different CO_2_ incubators at temperature settings of 37.0 and 37.4°C, and the results is consistent. The incubator was keep closed all throughout the EMF or non-EMF experiments to make sure that the conditions stable. Non-EMF groups were incubated in the same incubator in which the conditions were the same as for the exposed groups but without EMF.

### Patch-clamp Recordings

Whole-cell currents of granule neurons were recorded using a conventional patch-clamp technique. In 6–8 DIC cerebellar granule cells, transient *I*_Na_ are largely unclamped because of an event generated at a site electrotonically distant from the soma and prone to escape from clamp control, presumably the axon [47]. Therefore, we choose those cells that relatively isolated and only record currents without unclamped spike. Prior to current recordings, the culture medium was replaced with a bath solution containing (in mM): NaCl 145, KCl 2.5, HEPES 10, MgCl_2_ 1 and glucose 10 (pH adjusted to 7.4 using NaOH). Soft glass recording pipettes were filled with an internal solution containing (in mM): CsCl 145, HEPES 10, MgCl_2_ 2, and EGTA 5 (pH adjusted to 7.3 using CsOH). The pipette resistance was 5–6 MΩ after filling with the internal solution. Whole cell series resistances of 6–8 MΩ were routinely compensated by more than 70%. All recordings were performed at room temperature (23–25C°).

### Intracellular AA Assay

Intracellular AA levels in cerebellar GCs were measured as previously described [48], with minor modifications. Briefly, 1×10^5^ cells were plated in 35-mm dishes and grown to confluence. The cells were washed with 1 mL of bath solution and cerebellar GCs were exposed to 1 mT ELF-EMF for 10 min, 30 min, 60 min or 90 min. The media was then removed, and 0.3 mL of 0.45% NP40 (Sigma, St. Louis, MO, USA) was added to the plates. After a 5-minute incubation on ice, the lysate was removed from the plates and centrifuged for 5 minutes at 4°C. The supernatant was collected and assayed for AA levels using a direct rat AA ELISA kit (R&B, Yueyan Bio-Tech, Shanghai, China) according to the manufacturer’s instructions. The data represent the means of several experiments, as indicated in the figure legends.

### PGE_2_ Release Assay

PGE_2_ levels in the culture media were measured using highly sensitive enzyme-linked immunosorbent assay kits (R&B, Yueyan Bio-Tech, Shanghai, China) according to the manufacturer’s instructions. Briefly, 1×10^5^ cells were plated in 35-mm dishes and grown to confluence. The cells were washed with 1 mL of bath solution and were exposed to a 50 Hz, 1 mT EMF for 10 min, 30 min, 60 min or 90 min. The media were then collected and centrifuged for 5 minutes at 4°C. The supernatant was collected and assayed for levels of PGE_2_. The data represent the means of several experiments, as indicated in the figure legends.

### COX-1, COX-2 and PLA_2_ Enzyme Activity Assays

COX-1, COX-2 and PLA_2_ activities in cerebellar GCs were measured using highly sensitive, enzyme-linked immunosorbent assay kits (R&B, Yueyan Bio-Tech, Shanghai, China) according to the manufacturer’s instructions. Briefly, 1×10^5^ cells were plated in 35-mm dishes and grown to confluence. The cells were washed with 1 mL of bath solution and were exposed to 1 mT ELF-EMF for 10 min, 30 min, 60 min or 90 min. The media was then removed, and the cells were diluted with PBS (pH 7.2–7.4) until the cell concentration reached 1 million cells/ml. Following repeated freeze-thaw cycles, which result in cell damage and the release of intracellular components, the supernatant was collected by centrifugation for 20 min at 2000–3000 rpm and assayed for COX-1, COX-2 and PLA_2_ activities. The data represent the means of several experiments, as indicated in the figure legends.

### Phosphorylated Protein Kinase A Assay

The cells were lysed in HEPES-NP40 lysis buffer (20 mM HEPES, 150 mM NaCl, 0.5% NP-40, 10% glycerol, 2 mM EDTA, 100 µM Na_3_VO_4_, 50 mM NaF, pH 7.5, and 1% proteinase inhibitor cocktail) on ice for 30 min. After centrifugation, the supernatant was mixed with 2× sodium dodecyl sulfate loading buffer and boiled for 5 min. The proteins were separated on a 10% polyacrylamide gel, transferred to polyvinylidene difluoride membranes (Millipore, MA, USA), blocked with 10% nonfat milk and incubated at 4°C overnight with a rabbit polyclonal antibody against the phosphorylated form of the PKA catalytic subunits (1∶1000; Santa Cruz Biotechnology Inc., CA, USA) or a rabbit monoclonal antibody against GAPDH (1∶1000; Sigma). After extensive washing with TBST, the membrane was incubated with horseradish peroxidase-conjugated anti-mouse or anti-rabbit IgG (1∶10,000) (KangChen Bio-Tech, China) for 2 h at room temperature. Chemiluminescent signals were generated using a SuperSignal West Pico trial kit (Pierce, USA) and detected using a ChemiDoc XRS System (Bio-Rad Laboratories, Inc., CA, USA). The protein measurements were normalized to GAPDH and control/GAPDH as 1.0.

### Biotinylation Assay

Cell surface proteins were biotinylated according to the product instruction and previously described [49]. Briefly, the neurons were incubated with 0.25 mg/mL of Sulfo-NHS-SS-biotin (Thermo scientific, Rockford, USA) for 45 min at 4°C, and then the reaction is blocked with 50 mM Tris (pH 8.0) for 20 min at 4°C. Cells were lysed in HEPES-NP40 lysis buffer. Biotinylated proteins were pulled down with Streptavidin Agrose Beads (Thermo scientific, Rockford, USA) overnight at 4°C and washed for 4 times with lysis buffer. The bound proteins were eluted by the sample buffer and analyzed by western blotting.

### Immunocytochemistry

To detect changes in cPLA_2_ in cerebellar GCs following exposure to ELF-EMF, an immunocytochemical assay was performed using the following procedure. Isolated cortical neurons plated on coverslips were washed three times with 0.01 M PBS and then fixed with 4% PFA for 30 minutes, rinsed three times with PBS and pre-incubated for 1 hour in 6% normal donkey serum (v/v) in PBS plus 0.1% Triton X-100 at room temperature. The cells were then incubated with a rabbit anti-cPLA_2_ antibody (1∶100 dilution, Santa Cruz Biotechnology Inc., CA, USA) for 2 hours in a humidified air chamber. After incubation, the cells were rinsed three times with PBS and then further incubated with a secondary donkey anti-rabbit IgG tagged with FITC (1∶100 dilution, Jackson ImmunoResearch Laboratories, West Grove, PA) for 30 minutes at room temperature. The coverslips were then rinsed twice in PBS and mounted onto glass slides. For labeling of the Na_v_ 1.2 subunit, specific mouse monoclonal antibodies (from UC Davis/NINDS/NIMH NeuroMab Facility) were used as the primary antibody (at a working dilution of 1: 200). TRITC conjugated goat anti-mouse immunoglobulin G (1: 200 dilution, from Jackson Immunoresearch Laboratories, West Grove, PA) was used as the secondary antibody.

### siRNA design

Gene specific siRNA duplexes were designed to be homologous to Na_V1.2_ mRNA consensus sequence (GenBank accession number X03639). Primers for loop and 3′P-dT extensions contained 19-nt siRNA were commercially obtained (Invitrogen). The sense strand of the siRNA (5′-GGATATTGGTTCCGAAAAT-3′), homologous to nt 1965–1983 of the Na_V_ mRNA sequence, was chosen according to the recommendations by others (http://www.dharmacon.com/DesignCenter/DesignCenterPage.aspx). Restriction site Sac I was added to the 3′ tail of resulting molecule to facilitate ligating to LentiLox 3.7.

### Data Acquisition and Analysis

All currents were recorded using an Axopatch 200 B amplifier (Axon Instruments, Foster City, CA, USA) that was operated in voltage-clamp mode. A Pentium computer was connected to the recording equipment with a Digidata 1300 analog-to-digital (A/D) interface. The current was digitally sampled at 100 µs (10 kHz), and the current signals were filtered using a 5-kHz, five-pole Bessel filter. The currents were corrected online for leak and residual capacitance transients using a P/4 protocol. Data acquisition and analysis were performed using pClamp 8.01 software (Axon Instruments, Foster City, CA, USA) and/or Origin6.1 (MicroCal, Northampton, MA, USA). Statistical analysis was performed using Student’s *t*-test with non-paired or paired comparisons as relevant. The values are given as the means ± S.E.M., with *n* representing the number of cells tested. A value of *P*<0.05 was considered a significant statistical difference between groups. When multiple comparisons were made, the data were analyzed by a one-way ANOVA.

### Chemicals

All drugs used were purchased from Sigma-Aldrich (St. Louis, MO, USA) except for the fetal calf serum. The DMEM culture medium and antibiotic–antimycotic solution were obtained from Gibco Life Technologies (Grand Island, NY, USA). The arachidonic acid, 5,8,11,14-eicosatetraynoic acid, flufenamic acid, phorbol-12-myristate-13-acetate, AH6809 and AH23848 were first dissolved in DMSO and then diluted in extracellular solution to a final DMSO concentration of <0.2%, which by itself, did not affect *I*_Na_.
